# *JAK2* unmutated erythrocytosis: current diagnostic approach and therapeutic views

**DOI:** 10.1038/s41375-021-01290-6

**Published:** 2021-05-21

**Authors:** Naseema Gangat, Natasha Szuber, Animesh Pardanani, Ayalew Tefferi

**Affiliations:** 1 Division of Hematology, Department of Internal Medicine, Mayo Clinic, Rochester, MN USA; 2 Department of Hematology, Université de Montréal, Montréal, QC Canada

**Keywords:** Myeloproliferative disease, Myeloproliferative disease

## Abstract

*JAK2* unmutated or non-polycythemia vera (PV) erythrocytosis encompasses both hereditary and acquired conditions. A systematic diagnostic approach begins with documentation of historical hematocrit (Hct)/hemoglobin (Hgb) measurements and classification of the process as life-long/unknown duration or acquired. Further investigation in both categories is facilitated by determination of serum erythropoietin level (EPO). Workup for hereditary/congenital erythrocytosis requires documentation of family history and laboratory screening for high-oxygen affinity hemoglobin variants, 2, 3 biphosphoglycerate deficiency, and germline mutations that are known to alter cellular oxygen sensing (e.g., *PHD2, HIF2A, VHL*) or EPO signaling (e.g., *EPOR* mutations); the latter is uniquely associated with subnormal EPO. Acquired erythrocytosis is often elicited by central or peripheral hypoxia resulting from cardiopulmonary disease/high-altitude dwelling or renal artery stenosis, respectively; EPO in the former instance is often normal (compensated by negative feed-back). Other conditions associated with acquired erythrocytosis include EPO-producing tumors and the use of drugs that promote erythropoiesis (e.g., testosterone, erythropoiesis stimulating agents). “Idiopathic erythrocytosis” loosely refers to an otherwise not explained situation. Historically, management of non-PV erythrocytosis has been conflicted by unfounded concerns regarding thrombosis risk, stemming from limited phenotypic characterization, save for Chuvash polycythemia, well-known for its thrombotic tendency. In general, cytoreductive therapy should be avoided and phlebotomy is seldom warranted where frequency is determined by symptom control rather than Hct threshold. Although not supported by hard evidence, cardiovascular risk optimization and low-dose aspirin use are often advised. Application of modern genetic tests and development of controlled therapeutic intervention trials are needed to advance current clinical practice.

## Introduction

Erythrocytosis refers to either a true or apparent increase in hemoglobin (Hgb)/hematocrit (Hct); distinction requires familiarity with sex-, race- and altitude-adjusted normal values, together with an appreciation of extreme normal values that exceed the 95th percentile (2 SD) of reference range and attention to clinical factors associated with plasma volume depletion (relative erythrocytosis) [1, 2]. In 2008, the World Health organization (WHO) proposed Hgb and Hct diagnostic thresholds of 18.5 g/dl/52% and 16.5 g/dl/48% in Caucasian males and females, respectively, for investigation of polycythemia vera (PV) [3]. However, in 2016, the WHO classification system revised the Hct/Hct thresholds for PV diagnosis to 16.5 g/dL/49% in males and 16 g/dL/48% in females [4]. In a recent large population-based study, the incidence of erythrocytosis ranged from 0.3% to 3.4% based on the application of the WHO 2008 and 2016 criteria, respectively; importantly increased cardiovascular morbidity and mortality was reported only in the context of a higher Hgb threshold (WHO 2008) [3, 5].

The diagnostic workup of *JAK2* unmutated erythrocytosis lacks uniformity, and often includes investigations for rare hereditary conditions and several acquired entities known to be associated with secondary erythrocytosis [6]. In a substantial proportion (up to 70%) of patients, despite extensive testing, erythrocytosis remains uncharacterized and sometimes labeled as “idiopathic”, which is often a diagnosis of exclusion [7–9]. The complexity and variability of the decision-making processes involved in managing erythrocytosis were underscored by an international survey of 134 myeloproliferative neoplasm (MPN) physicians evaluating standard practice in erythrocytosis [10]; in this regard, while first-line assessments were relatively consistent (*JAK2*, EPO testing), second-line investigations were markedly heterogeneous. Equally variable were treatment practices, including the decision to initiate daily aspirin therapy and/or phlebotomy, as well as the desired Hct target, ranging from 48–56%. Some of this variability in management stems from unsubstantiated concern regarding thrombosis risk. The particular issue was further highlighted in a recent retrospective study where as many as one-third of patients with secondary erythrocytosis were subjected to bone marrow examination and 42% to periodic phlebotomy [11]. Herein, we outline our systematic diagnostic and therapeutic approach in *JAK2* unmutated erythrocytosis; our review also includes illustrative cases and a discussion on underlying pathogenetic mechanisms.

## Illustrative cases

### Case 1: Erythropoietin receptor (EPOR) mutation

A 48-year-old Caucasian lady, an elementary school teacher from Minnesota, was noted to have erythrocytosis while undergoing a kidney donor evaluation. Hgb and Hct were 19.4 g/dl and 57%, respectively, with normal leukocyte and platelet count. The patient had a history of persistent erythrocytosis with Hgb/Hct of 19.3 g/dl/58%, documented two decades prior to her diagnosis. Her medical history was significant for hypertension, optimally controlled with lisinopril; tobacco use, vascular events, or microvascular symptoms were absent in her medical history. Importantly, both her mother and brother were previously noted to have erythrocytosis. Initial laboratory evaluation included EPO (1.1 mIU/mL; normal reference range 2.6–18.5) and peripheral blood *JAK2* mutational screen (wild-type for both exons 12 and 14). Based on family history and subnormal EPO, *EPOR* mutation screen was pursued and revealed a heterozygous c.1316G>A, p.(Trp439*) mutation in exon 8 that caused a premature stop codon and truncated the 70 C-terminal amino acids resulting in a gain of function [12]; the altered C-terminal increases EPOR stability at the cell surface resulting in pre-activation of both receptor and constitutive JAK2 signaling similar to what is seen in PV [13]. This *EPOR* mutation has been shown to be associated with autosomal dominant erythrocytosis [14] and also reported as a de novo alteration [15, 16]. Taken together, this information supported a diagnosis of autosomal dominant hereditary or familial erythrocytosis type 1. Most patients with *EPOR* mutations are asymptomatic; with isolated cases experiencing cardiovascular complications and deep vein thrombosis [17]. While phlebotomy may benefit some patients in alleviating symptoms, it does not appear to have a protective effect with regards to cardiovascular events [18]. In terms of management, optimal hypertension control with lisinopril was emphasized and low-dose aspirin initiated. Since our patient was asymptomatic, routine phlebotomy was not recommended, however, phlebotomy prior to kidney donation was a consideration.

### Case 2: Secondary erythrocytosis associated with cerebrovascular malformations

A 17-year-old Caucasian male, a high school student from Missouri was referred for evaluation of unexplained erythrocytosis. He reported a myriad of symptoms including nausea, vomiting, abdominal pain, weight loss, fatigue, headaches, cold hands and feet with numbness and tingling, hyperhidrosis, palpitations, and pruritus. He was a non-smoker without illicit drug or hormone use. Family history was unremarkable. On physical examination, no cutaneous, abdominal or neurological abnormalities were noted. A review of serial blood counts showed a consistent elevation in Hgb >16.5 g/dl, with peak Hgb/Hct of 18.3 g/dl/52.4% and normal leukocyte and platelet counts. PV was excluded with a negative *JAK2V617F*/exon 12 mutation screen and elevated EPO at 22.6 mIU/mL (normal range; 2.6–18.5 mIU/mL). Bone marrow aspirate/biopsy was without features of a MPN. Hereditary erythrocytosis evaluation did not reveal high-affinity Hgb variants or mutations in 2,3-Bisphosphoglycerate Mutase (*BPGM)*(exons 1–4), *EPOR* (exon 8), or oxygen‐sensing pathway proteins, including hypoxia-inducible factor 2 α (HIF2A) encoded by endothelial PASS domain protein 1 *(EPAS1)* (exons 9 and 12), prolyl hydroxylase 2 (PHD2) encoded by egl-9 family hypoxia-inducible factor 1*(EGLN1)*(exons 1-5), von Hippel Lindau (*VHL)* (three coding exons and intron/exon boundaries). Chest/abdominal imaging, arterial blood gas, transthoracic echocardiogram with shunt study, urinary catecholamines/metanephrines were unremarkable. Interestingly, MRI brain/spine revealed multiple cerebrovascular malformations; both developmental venous anomalies and cavernous hemangiomas in the frontal and temporal regions. Accordingly, the cerebrovascular malformations were hypothesized to be a plausible link to the patient’s erythrocytosis. Surgical intervention was not feasible due to the presence of multiple anomalies. He was initiated on phlebotomies with marked improvement in symptoms; thereafter, need for periodic phlebotomies was determined by symptoms rather than a target Hct value. This previously published case underscores the importance of a systematic diagnostic approach to *JAK2* unmutated erythrocytosis [19].

### Case 3: “Idiopathic” erythrocytosis (erythrocytosis not otherwise specified)

Sixty-six-year-old lady from Minnesota with history of mild asthma, recurrent generalized anxiety, and episodic hypertension with possible right subclavian steal, was referred for evaluation of erythrocytosis. Complete blood count revealed Hgb of 19.3 g/dL, Hct of 58%, leukocytes 6.5 × 10^9^/l, and platelet count of 349 × 10^9^/l. Historical blood count review noted Hgb of 18.6 g/dl four years prior with progressive rise in Hgb >19 g/dl. Family history was unremarkable, and the patient was a non-smoker, without history of thrombosis, or aquagenic pruritus. Her dominant symptoms included severe headaches and fatigue. Physical exam revealed hypertension, normal oxygen saturation, without palpable hepatosplenomegaly. EPO level was in the normal range at 13.9 mIU/mL and *JAK2V617F*/exon 12 mutation analyses were negative. Bone marrow examination lacked features of MPN. Abdominal ultrasound did not reveal renal or hepatic lesions or evidence for renal artery stenosis. CT head was without intracranial abnormalities. Sleep apnea was excluded by overnight oximetry. Monoclonal protein studies and pheochromocytoma evaluation with urinary catecholamines/metanephrines were also obtained and unrevealing. In addition, hereditary erythrocytosis workup including high-oxygen affinity Hgb variants, *EPOR, BPGM, VHL, PHD2, HIF2A* mutation testing was unrevealing. In the absence of a specific etiology for erythrocytosis, our working diagnosis was “idiopathic” erythrocytosis or erythrocytosis not otherwise specified. She was recommended aspirin 81 mg daily and initiated on phlebotomy due to her symptoms and labile hypertension. The need for periodic phlebotomy was based upon symptoms. At six-month follow-up, Hgb remained below 16 g/dl without phlebotomy, along with resolution of headaches and hypertension. We recommended close monitoring of blood counts every 3–6 months.

## Pathogenetic mechanisms in hypoxia-induced secondary erythrocytosis

The HIF pathway regulates erythropoiesis and EPO production within renal peritubular cells, in an oxygen-dependent manner [20–22]. HIF transcription factor is a heterodimer with alpha and beta subunits, the latter is constitutively expressed, while hypoxia affects the function of the former. HIF-A has three known isoforms (HIF-1A, HIF2A, HIF-3A), amongst which HIF2A is mainly involved in regulation of EPO synthesis; *HIF2A* knockout mice demonstrate a hypocellular marrow and anemia as a result of inadequate EPO production [23]. In the presence of oxygen, HIF2A undergoes hydroxylation at two critical proline residues, Pro405 and Pro531, mediated by PHD2 [24, 25], following which it undergoes degradation by the ubiquitin proteasomal pathway, a process mediated by VHL, a tumor suppressor protein, serving as the substrate recognition component of an E3 ubiquitin ligase complex [26]. In addition, a 2-oxoglutarate dependent oxygenase, factor inhibiting HIF (FIH), catalyzes hydroxylation of a specific arginine residue within HIF which inhibits HIF binding to p300, a transcriptional co-activator [27]. The end result is reduced transcriptional activation of *EPO*; a HIF target gene.

Conversely, under hypoxic conditions, PHD2 enzymatic activity is reduced, resulting in diminished hydroxylation and degradation of HIF2A, in other words stabilization of HIF2A following which the HIF complex binds hypoxia responsive elements within the *EPO* gene and turns on its transcription [28–30]. This constitutes the pathogenetic basis for hypoxia-induced acquired erythrocytosis associated with chronic obstructive pulmonary disease (COPD), cyanotic heart disease with right to left shunt, and high-altitude habitat. Amelioration of tissue hypoxia reverses the process resulting in compensated normal EPO in such conditions, consistent with the assertion that regulation of erythropoiesis by HIF is exquisitely sensitive to oxygen levels [31, 32]. In addition, HIF2A expression is also controlled by iron regulatory proteins 1 and 2 (Irps) via its iron-responsive elements, and deletion of *Irp1* in murine studies has been found to increase HIF2A expression, which in turn stimulates EPO, leading to erythrocytosis [33].

## Pathogenetic mechanisms in hereditary erythrocytosis

### Germline VHL/HIF2A/PHD mutations

**Fig. 1:**
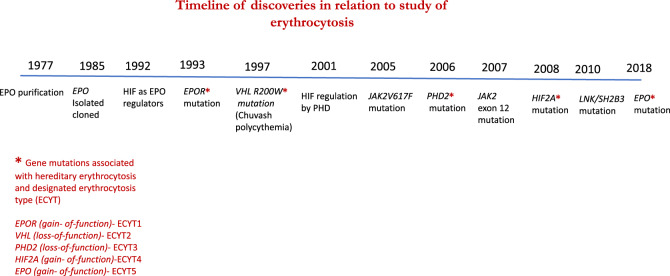
Timeline of discoveries in relation to the study of erythrocytosis.

Germline mutations in the oxygen-sensing *(VHL-HIF2A-PHD*) pathway genes are relatively rare but when present may result in erythrocytosis with elevated or inappropriately normal EPO [34–43]. It is to be noted that interrogation of this pathway is an evolving area of investigation with recent identification of novel zinc finger domain *PHD2* mutations and splicing mutations in *VHL* [44, 45]. In chronological order (Fig. 1), in 1997, Chuvash polycythemia (CP), an autosomal recessive condition with homozygous VHL (R200W) mutation was initially described in Chuvashia (Russia) and subsequently noted in the Italian island of Ischia and worldwide [34, 35, 46, 47]. Functionally, VHL–HIF2A interaction is disrupted with impaired ability of VHL to target hydroxylated HIF2A for proteasomal degradation, resulting in increased HIF2A and EPO levels under normoxic conditions [34]. In addition, mutant VHL exhibits altered affinity for suppressor of cytokine signaling 1, impeding formation of a heterodimeric E3 ligase involved in targeting phosphorylated JAK2 for ubiquitin-mediated proteasomal degradation [48]. Accordingly, erythroid progenitors in affected patients also displayed hypersensitivity to EPO, which is an inherent feature shared by PV. In contrast to what is seen with the autosomal dominant “VHL syndrome”, malignant and benign tumors, including spinocerebellar hemangioblastomas, pheochromocytoma and renal cell carcinoma are not seen in patients with CP, reflecting the differential impact of the mutation in terms of HIF regulation [49]. On the other hand, erythrocytosis seldom accompanies “VHL syndrome” and is typically mediated by ectopic EPO production by tumor [50, 51].

In 2006, a *PHD2* mutation (P317R) was implicated in a family with erythrocytosis; EPO levels were inappropriately normal, pointing toward secondary erythrocytosis [37]. The loss of function *PHD2* mutations (P317R and the P371H variants), affect the catalytic rate and substrate-binding of PHD2, thereby hindering HIF hydroxylation. These mutations are not typically associated with tumors except for a patient with a recurrent extra-adrenal paraganglioma harboring the *PHD2* H374R mutation [39].

In 2008, a heterozygous missense gain of function *HIF2A* mutation, located near the primary site of HIF hydroxylation (Pro531) was discovered upon investigation of a family with erythrocytosis; the particular mutation results in stabilization of HIF2A due to impaired hydroxylation [40]. *HIF2A* mutations may be associated with neuroendocrine tumors with a recent genotype/phenotype study describing the clinical spectrum of HIF2A mediated diseases (*n* = 66): class 1a (*n* = 6, sporadic) with pheochromocytoma, paraganglioma, somatostatinoma and erythrocytosis; class 1b (*n* = 12; 1 familial, 11 sporadic) with pheochromocytoma, paraganglioma and erythrocytosis; class 1c (*n* = 20; all sporadic) with pheochromocytoma and paraganglioma, and class 2 (*n* = 28; 9 familial, 6 sporadic) with erythrocytosis alone. Each class was found to be driven by a unique set of non-overlapping mutations with class 1 mutations typically located between amino acid residues 529 and 532, which contain Pro531, whereas, class 2 mutations were found exclusively between residues 533 and 540. These findings highlight the differential impact of *HIF2A* mutations on HIF2A-VHL interaction with higher HIF2A levels implicated in tumorigenesis (class 1 disease) as opposed to slight increases in HIF2A activity that are sufficient to induce erythrocytosis in class 2 disease [52].

### EPOR mutation

EPO binds to its receptor (EPOR) on the erythroid progenitor surface, which is part of the cytokine class I receptor superfamily; the receptor subsequently undergoes dimerization and activates JAK2, which in turn leads to tyrosine phosphorylation of its distal region [53]. EPO-induced JAK2 activation leads to intracellular activation of the Ras/mitogen-activated protein kinase, phosphatidylinositol 3-kinase/Akt pathways, and signal transducer and activator of transcription (STAT 1, 3, 5A, 5B), which turn on numerous target genes that promote red cell survival, proliferation and maturation [53]. In essence, EPO in synergy with several growth factors (SCF, GM-CSF, 1L-3, and IGF-1) enhances red cell survival by inhibition of apoptosis together with maturation and proliferation of erythroid progenitor cells. Germline heterozygous nonsense and frameshift mutations in exon 8 of *EPOR* have been described causing truncation of the C-terminal distal portion of the receptor which contains several tyrosine residues that serve as docking sites for SHP1, SOCS3 that are negative regulators of EPO signaling [14, 54–56]. This results in hypersensitivity to EPO via excessive activation of the EPO receptor [57–59].

Recently, mutations in the *EPO* gene itself have been identified in relation to familial erythrocytosis whereby a single base pair deletion in exon 2 caused a frameshift that truncated translation of the main *EPO* messenger RNA (mRNA), but converted a typically noncoding mRNA, which was transcribed from an alternative promoter within intron 1, to produce excess functional EPO mainly through the liver [60]. In addition, in a five-generation kindred with erythrocytosis, a novel heterozygous 5′UTR *EPO* variant has been newly discovered; the mutated 5′UTR of *EPO* augments interaction with HIF2, leading to increased production of EPO [61].

### Altered oxygen affinity (high-oxygen-affinity Hgb variants and 2, 3 BPGM deficiency)

Structurally, Hgb is a tetramer, comprising of two alpha and beta globin subunits (an α_1_β_1_ dimer and an α_2_β_2_ dimer in Hgb A) with two conformationally stable states; the relaxed (R), high-oxygen affinity state and tense (T), low-oxygen affinity state [62, 63]. Oxygen binding to Hgb subunits demonstrates cooperativity, resulting in a sigmoidal oxygen dissociation curve which is left-shifted (low p50) with high-affinity variants [64]. The *R*–*T* transition is impacted by mutations in critical regions of the globin chain in high-affinity variants. Oxygen delivery is compromised at the tissue capillary level, resulting in hypoxia which serves as a stimulus for EPO production and subsequent erythrocytosis. Almost, 100 high-oxygen affinity Hgb variants have been reported [65]; a review of the Mayo Clinic Hgb variant database (1974-2018) identified 762 patients with 80 distinct variants (61 β, 20 α) [66]. These mutations were mostly missense impacting the heme pocket, α1β2 contact sites, 2, 3 BPG binding sites and C-terminal conformation stabilization regions, with the most common variants being Hb Tarrant (α chain variant) and Hb Malmo (β chain variant) [66]. Only one-third of high-affinity variants give rise to erythrocytosis, likely a result of either low‐level expression of the variant or concomitant hemolysis [65, 67]. Within red cells, 2, 3, BPGM catalyzes conversion of 1, 2 BPG to 2, 3 BPG, the latter binds deoxy Hgb tetramer and allosterically converts it to a low-oxygen affinity state, prompting release of oxygen. However, with 2, 3 BPGM deficiency, conversion of 1, 2 BPG to 2, 3 BPG is impaired, which enables the Hgb tetramer to assume a high-oxygen affinity state. Erythrocytosis resulting from 2, 3 BPG deficiency is relatively uncommon with limited cases described in the literature [68, 69].

## Diagnostic approach

In clinical practice, hematology referrals for erythrocytosis are triggered by Hgb/Hct level above 16.5 g/dl/49% and 16 g/dl/48% in Caucasian males and females, respectively, and should be confirmed by at least two separate blood counts evaluated at different time points. This should be followed by distinguishing true from apparent erythrocytosis; the former is a result of either a clonal MPN, as in PV, or a non-clonal process resulting in secondary erythrocytosis. It is also important to recognize inapparent (masked) erythrocytosis in which an increase in red cell mass (RCM) is accompanied by a concomitant increase in plasma volume and therefore masked by a normal Hgb/Hct. Similarly, in situations with normal Hgb/Hct and concomitant iron deficiency, PV is often underrecognized. Conversely, an apparent erythrocytosis may result from either a reduction in plasma volume (relative erythrocytosis) or extreme high normal values which exceed the 95th percentile of sex-, race-and altitude-adjusted normal values. RCM measurement is no longer available at our institution; however, upon evaluation of its performance and practical utility in a total of 105 patients with erythrocytosis; elevated RCM was seen in 76%, 20%, 21%, and 57% with PV, secondary erythrocytosis, apparent erythrocytosis, and essential thrombocythemia, respectively. In the particular study RCM significantly correlated with both Hct and Hgb levels (*p* < 0.001). Hgb/Hct values below which an elevated RCM was not observed were 16/48% for males and 13/39% for females. In contrast, values >19.5/58% for males and 17.5/53% for females were almost always associated with increased RCM [70]. In other words, RCM measurement was found to be suboptimal in distinguishing PV from other causes of erythrocytosis and failed to offer any additional diagnostic value. Furthermore, obesity is a confounding factor that hinders accurate interpretation of RCM values [71].

During the evaluation of erythrocytosis, PV should always be considered and the diagnosis excluded by the absence of a *JAK2* mutation (*V617F* exon 14 and exon 12) [72, 73]; also helpful in this regard is serum EPO especially in cases of *JAK2*V617F-negative but exon 12 mutated PV, where serum EPO levels are often subnormal [74]. If PV is suspected clinically or EPO is subnormal with the absence of *JAK2*V617F, a bone marrow examination is recommended in order to identify histological features associated with MPN. Additional testing for MPN associated mutations; *CALR* and *SH2B3/LNK*, albeit rarely found in PV, may be pursued [75–79]. Though predating molecular designations and not uniformly performed, in centers where available, bone marrow erythroid progenitor cultures may be a useful adjunct to rule-out endogenous erythroid proliferation [80, 81]. According to a seminal study on the subject, endogenous erythroid colonies were noted in the vast majority of PV (43 of 46) and in 0 of 17 secondary erythrocytosis cases [82], substantiating the enduring value of this test.

**Fig. 2:**
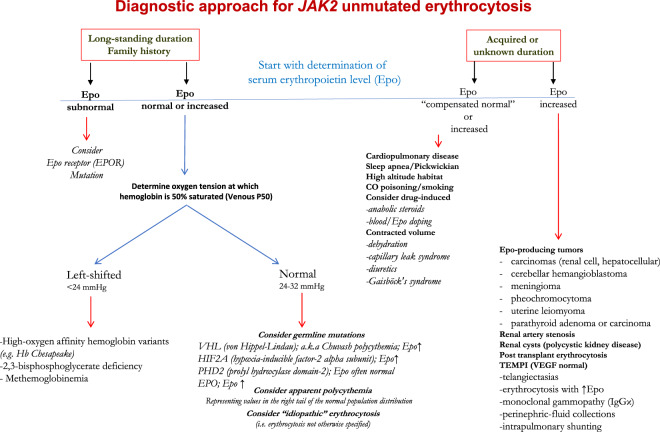
Diagnostic approach for *JAK2* unmutated erythrocytosis.

Once PV has been conclusively ruled out, we entertain the possibility of either hereditary or acquired erythrocytosis and commence our workup by reviewing prior blood counts when available in order to determine the duration of erythrocytosis. Figure 2 reviews our diagnostic algorithm which hinges upon duration of erythrocytosis (if known) and EPO levels.

### Workup for suspected hereditary erythrocytosis

Hereditary erythrocytosis is suspected in children and young adults with long-standing erythrocytosis, particularly with a positive family history [83, 84]. A subnormal serum EPO level is suspicious for the presence of an *EPOR* mutation, as illustrated in Case 1. On the other hand, if serum EPO is normal or elevated, we proceed with venous p50 measurement (oxygen tension at which Hgb is 50% saturated). Venous p50 may also be calculated from venous blood gas using the following mathematical formula https://www.medsci.org/v04/p0232/ijmsv04p0232s1.xls [85].

A left shift of the oxygen dissociation curve, that is venous p50 < 24 mmHg may result from high-oxygen affinity Hgb, defective 2,3 BPG mutase causing 2, 3 BPG deficiency or methemoglobinemia [66]. Detection of high-oxygen-affinity Hgb variants is accomplished by cation exchange HPLC, capillary electrophoresis, or mass spectrometry. Since HPLC is normal in one-third of cases, sequencing of *HBB*, *HBA1, HBA2* genes is often necessary.

Mutations in the oxygen-sensing pathway (*VHL, PHD2, HIF2A*) do not affect the venous p50 measurement and EPO is either elevated or inappropriately normal; in this regard, it is important to note that serum EPO might be impacted by phlebotomy. Sequencing studies for *VHL* (exons 1–3), *PHD2* (exons 1–5), *HIF2A* (exons 9 and 12) are performed. Our Mayo clinic laboratory experience lends to the rarity of the above entities; of 1192 cases tested for hereditary erythrocytosis only 143 (12%) cases had identified abnormalities of which 85 were pathogenic or likely pathogenic mutations (inclusive of alpha and beta high-oxygen affinity Hgb) and 58 variants of unknown significance [66]. Phenotypically, the majority of patients with high-oxygen affinity Hgb variants are asymptomatic [67].

### Diagnostic approach in acquired erythrocytosis

A workup for acquired erythrocytosis warrants a thorough medical evaluation with attention to place of residence, tobacco use, along with a careful review of medications both prescription and supplements particularly testosterone, androgen, erythropoietin, diuretics, and certain antidiabetic agents (sodium-glucose cotransporter 2 inhibitors; e.g., canagliflozin). Physical examination should focus on identifying hypoxia, cardiopulmonary disease, telangiectasias, cushingoid or virilization features, and abdominal masses raising concern for renal or hepatic tumors. Initial testing should include arterial blood gas, overnight oximetry, and abdominal ultrasound to assess for renal/hepatic tumors/renal artery stenosis. Further investigations are dictated by clinical findings; in the instance of neurological symptoms, brain imaging is of utility as in Case 2. An echocardiogram with shunt study is indicated in the presence of relevant cardiac findings. In females, myomatous erythrocytosis syndrome should be kept in mind, particularly since erythrocytosis is often masked by menorrhagia [86]. Rare acquired disorders to consider include the monoclonal gammopathy driven TEMPI syndrome which is characterized by telangiectasias, elevated serum EPO (which might be >5000 mIU/ml) and erythrocytosis, monoclonal gammopathy, perinephric fluid collections and intrapulmonary shunting [87, 88]; erythrocytosis and telangiectasis are often the initial manifestation, therefore obtaining monoclonal protein studies might provide diagnostic clues to this entity. Similarly, erythrocytosis along with thrombocytosis may be a part of polyneuropathy, organomegaly, endocrinopathy, M‐protein, skin changes syndrome [89].

### Idiopathic erythrocytosis

In a high proportion (70%) of patients with erythrocytosis, a specific etiology remains elusive despite extensive testing; such cases are often referred to as “idiopathic” or “not otherwise specified” [7, 55]. In rare instances*, SH2B3/LNK* exon 2 mutations or polymorphisms have been reported in patients with unexplained erythrocytosis, with subnormal serum EPO levels, and absence of *JAK2, MPL,* and *EPOR* mutations [90, 91]. For instance, *LNK* mutations were detected in 6 of 112 (5.3%) of patients with *JAK2* negative erythrocytosis, assumed to be idiopathic [92]. In patients with clinical suspicion of hereditary erythrocytosis, aberrant functioning of proteins involved in the oxygen-sensing pathway (PHD2-HIF2A-VHL-EPO) and erythropoiesis have a high likelihood of being implicated, hence expanded gene/exome sequencing should be offered if available on a research basis in order to uncover novel variants/polymorphisms [7]. Recently, pathogenic mutations in the *PIEZO1* gene were noted in up to 4% of individuals with idiopathic erythrocytosis, in association with clinical or biological manifestations of hereditary xerocytosis (HX)(iron overload, splenomegaly, hemolysis, decreased venous p50) [93]. In a large series of patients with HX and *PIEZO1* mutations, 68% were not anemic; moreover, 7 adults had Hgb >16 g/dl, with two patients known to have erythrocytosis [94]. Functionally, Piezo1-HX affects red cell energy metabolism and glycolysis, resulting in reduced BPG levels, conferring a high-oxygen affinity state, which explains the basis for erythrocytosis in this disorder [95]. Another rare entity to consider particularly in children with unexplained erythrocytosis are *SLC30A10* mutations, which occurs in association with hypermanganesemia, parkinsonism, and hepatic cirrhosis [96].

Continued monitoring of cases with unexplained erythrocytosis is advised, especially in those where information on *JAK2* mutation is not available. Not surprisingly, in comparison to patients with PV, those with idiopathic erythrocytosis were more likely to be males and display lower white and platelet count, higher EPO levels, lower lactate dehydrogenase levels, absence of palpable splenomegaly or thrombosis [11, 97]. In the particular study, phlebotomy led to elevation in leukocyte and platelet count in PV but not in idiopathic erythrocytosis [97]. Another report comparing 145 patients with idiopathic erythrocytosis and PV also confirmed a lower incidence of thrombosis and bleeding with idiopathic erythrocytosis [98].

## Management

**Fig. 3:**
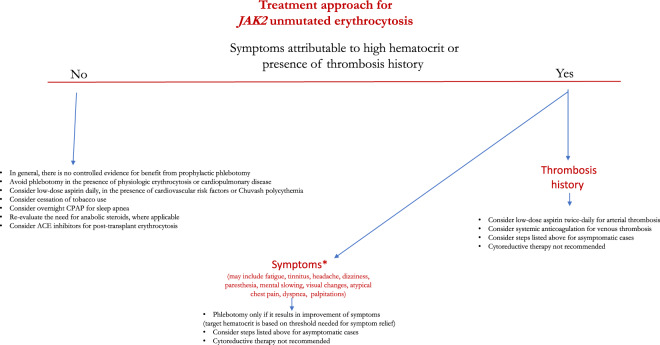
Treatment approach for *JAK2* unmutated erythrocytosis.

A dearth of compelling evidence defining optimal management strategies for *JAK2* unmutated erythrocytosis stems from the heterogeneity in associated hereditary and acquired disorders. Hence, it is common practice to inadvertently extrapolate outcomes from studies conducted in PV patients or follow consensus guidelines [99, 100]. In order to formulate our recommendations that have been summarized in Fig. 3, we reviewed the relevant literature pertaining to secondary erythrocytosis associated with common acquired and hereditary conditions (Table 1). Data regarding thrombosis risk in acquired secondary erythrocytosis has been conflicting; while the majority of studies do not support an increased thrombotic risk [101–103], two recent studies have implied the contrary [5, 11]. In a noteworthy large population-based series, erythrocytosis defined by higher Hgb/Hct thresholds, as per the 2008 WHO criteria, was associated with higher cardiovascular morbidity and mortality [5]; however, details of conditions associated with erythrocytosis were not specified but a high incidence (38%) of clonal hematopoiesis and cardiovascular risk factors were reported accounting for a higher incidence of cardiovascular events. In a second smaller series of 35 patients with secondary erythrocytosis, thrombosis rates at or prior to diagnosis were similar to those reported in PV, which is likely explained by the clustering of cardiovascular risk factors in those with secondary erythrocytosis [11].

**Table 1:** Selected practically relevant studies focusing on management issues in *JAK2* unmutated erythrocytosis.

Condition predisposing to erythrocytosis (Incidence of erythrocytosis)	Study/Reference	Noteworthy findings
COPD (5.9–18.1%)	Prognostic value of hematocrit in severe COPD receiving long-term oxygen therapy (retrospective observational study) Chambellan et al., Chest [125]	- 2524 COPD pts with mean HCT 45.9 ± 7.0% in men and 43.9 ± 6.0% in women. - HCT an independent predictor of survival, hospital admission rate, and cumulative duration of hospitalization. - 3-year survival 24% when HCT < 35%, 59% with HCT 50–54%; and 70% when HCT ≥ 55% (*p* < 0.001).
	Hemoglobin levels and long-term survival in COPD. (retrospective study) Kollert et al. [107]	- 309 COPD pts with chronic respiratory failure under optimized therapy, including oxygen and non-invasive ventilation. Excluded pts on phlebotomy. - 46 (14.9%) anemic (Hb < 12 g/dL in females, Hb < 13 g/dL in males), 56 (18.1%) polycythemic (Hb ≥ 15 g/dL in females, Hb ≥ 17 g/dL in males), or 207 (67%) normocythemic with median survival 29, 112 and 51 months (*p* = 0.008). (values determined prior to oxygen therapy). - Hb ≥ 14.3 g/dL in females, and ≥15.1 g/dL in males independently associated with longer survival.
	Pulmonary embolism in chronic hypoxemic patients. (prospective study) Ristic et al., Med Glas (Zenica) [126]	- 362 pts with severe COPD exacerbation or raspatory failure, with D-dimer ≥500 µg/l referred for Doppler ultrasound and CT. - Group 1 (100 pts with erythrocytosis) vs group II (262 without erythrocytosis). - Higher pulmonary embolism in group 1 vs 2 (39% vs 11.1%)
	Prevalence of VTE in COPD with erythrocytosis. (retrospective case-control study) Nadeem et al. Clinical and Applied Thrombosis/Hemostasis [101]	- COPD with erythrocytosis, HCT ≥ 50 (*n* = 86) vs age, sex-matched COPD without erythrocytosis, HCT < 50 (*n* = 86). - No difference in VTE ;17 (19.8%) cases vs 12 (14%) controls, *p* = 0.42 - Trend toward higher incidence of idiopathic VTE in cases (*n* = 10 vs 4, *p* = 0.16) - Similar BMI, smoking, cancer, OSA with differences in hematocrit, oxygen use, pulmonary HTN. Phlebotomy data not provided
	Incidence of cardiovascular and thrombotic events in secondary polycythemia. (retrospective study) Mao et al. [109]	- Impact of phlebotomy on prevalence of arterial/venous thrombosis in COPD with erythrocytosis (*n* = 115). - Thrombosis in 11/35 (31.4%) phlebotomized vs 26/116 (22.4%) in non-phlebotomized (*p* = 0.28). - Amongst phlebotomized pts, thrombosis in 4/16(25%) with HCT < 52% vs 7/19(36.8%) in those with higher HCT (*p* = 0.45)
	Secondary polycythemia and perioperative hemorrhage or thrombosis. (retrospective case-control study Lubarsky et al. [102]	- COPD with erythrocytosis, Hb > 16 g/dl (16.2–20.1 g/dl) (*n* = 100) vs age, sex, surgery, ASA physical status-matched controls without erythrocytosis. - No thrombosis in erythrocytosis group vs 3 events in controls. - Significantly less transfusion requirement in erythrocytosis group vs controls (*p* < 0.025).
	Symptomatic and pulmonary response to acute phlebotomy in secondary polycythemia. (Double-blind clinical trial) Dayton et al. [110]	- 11 pts with hypoxic lung disease associated erythrocytosis, HCT > 54 underwent phlebotomy vs 8 pts as controls (sham procedure). - 8/11 phlebotomized patients with symptom (dyspnea, fatigue, headache) improvement within 24 h and lasted > 7 days in 5 pts, particularly if HCT > 60 vs no improvement in controls (*p* < 0.005). - No objective improvement in airway obstruction, gas exchange or exercise tolerance.
Cyanotic congenital heart disease	Risk of stroke in adults with cyanotic congenital heart disease. (observational study) Perloff et al. [104]	- 112 adults with cyanotic heart disease excluding those with independent risk factors for stroke were observed for 748 pt-years. - 2 groups, (i) compensated (HCT 46–72%, absent/mild symptoms, iron replete with phlebotomy at intervals > 1 yr if symptoms) (*n* = 101) vs (ii) decompensated (HCT 61.5–75%), iron deficiency, marked/severe symptoms with phlebotomy every 3–6 months (*n* = 11). - None of the pts in group (i) had cerebral arterial thrombosis, vs one pt in group (ii) with amaurosis fugax.
	Cerebrovascular events in adults with cyanotic congenital heart disease (retrospective study) Ammash et al. [104]	- 162 adults with cyanotic congenital heart disease; Group I (*n* = 140) no history of cerebrovascular event after age 18 years, Group II (*n* = 22) well documented cerebrovascular event (TIA (*n* = 19), reversible ischemic neurologic deficit *n* = 4, infarct, *n* = 6). - 46/162 (28.4%) underwent phlebotomy, with increased risk of events after phlebotomy (35/140 in Group I vs 11/22 in Group 2, *p* = 0.016). - Strong association of iron deficiency and/or microcytosis with cerebrovascular events (11/41; *p* = 0.004).
	Hydroxyurea for secondary erythrocytosis in cyanotic congenital heart disease. (case series) Reiss et al. [113]	- 4 pts with symptomatic secondary erythrocytosis and cyanotic congenital heart disease. - 2 pts with recent TIA or stroke, one with extreme fatigue/dyspnea and one with extreme exhaustion following phlebotomy. - Median hydroxyurea use: 15 months; symptomatic improvement, but myelosuppression in 2 pts and thrombocytopenia in 1 pt which required dose reduction. 1 pt with TIA on hydroxyurea. Minimal increase in HbF.
Post-renal transplant erythrocytosis (8-15%)	Enalapril for erythrocytosis after renal transplant. (randomized double-blind study) Beckingham et al. [116]	- 2.5 mg of enalapril daily (*n* = 15) or placebo (*n* = 10) for 4 months. - Hematocrit decreased from 52.7 to 47.1 at 1 month and 46.1 after 4 months with enalapril, no change with placebo (*p* = 0.004). No change in erythropoietin level. - No phlebotomy or thrombosis.
	Effects of theophylline on erythrocytosis after renal transplant. (prospective study) Bakris et al. [118]	- Pts with post-renal transplant erythrocytosis (*n* = 8) vs normal controls (*n* = 5). - 8 weeks of theophylline, EPO significantly reduced in transplant pts (60 to 9 units; *p* < 0.05) and controls (6.9 to 4.7 units; *p* < 0.05). - Hematocrit reduced in transplant pts (0.58 to 0.46; *p* < 0.05) and controls (0.43–0.39; *p* < 0.05). Requirement of weekly phlebotomy eliminated.
	Comparison of enalapril and losartan on post-renal transplant erythrocytosis. (prospective randomized study) Yildiz et al. [117]	- 27 pts treated with enalapril 10 mg/day (*n* = 15), vs losartan 50 mg/day (*n* = 12) for 8 weeks. - Hemoglobin significantly decreased with both losartan (17.1–15.9 g/dl, *p* = 0.01) and enalapril (17.4–14.9 g/dl, *p* = 0.001). greater decrease with enalapril (−3.26 vs −1.70, *p* = 0.05). - Among the responders who discontinued treatment, there was a trend for longer time to relapse with losartan (7.38 months) compared with enalapril (2.75 months) (*p* = 0.11).
Testosterone therapy and erythrocytosis	Prevalence and management of secondary erythrocytosis in transgenders on testosterone. (retrospective study) Oakes et al. [122]	- 234 pts, mean pre-testosterone hemoglobin 13.5 g/dL and hematocrit 40.3%. Mean hemoglobin peak 15.7 g/dl, hematocrit 47.2% at an average of 21 months post therapy. 23.5% pts with hematocrit > 50%, and 8.5% hemoglobin > 17.5 g/dL. Only one thrombotic event. - Dose reduction in 14.5% with erythrocytosis, no phlebotomy. - 88.9% of patients with erythrocytosis had received testosterone cypionate
Chuvash polycythemia (CP)	Thrombosis in CP. (prospective study) Sergueeva et al. [105]	- CP pts matched by age, sex and place of residence (n = 128). - CP pts with lower blood pressure, body mass index, white count, but more smokers than controls. - New thrombosis in CP pts 0.031 events/pt/year (34 arterial+venous events vs 3 arterial events in controls), history of phlebotomy but not hematocrit predictor of thrombosis. - 9 (7.0%) deaths in CP pts (median age; 54 years) compared to 2 (1.6%) controls (median age at death; 81 years) (*p* = 0.058). All deaths related to thrombosis.
Congenital erythrocytosis	Thrombotic risk in congenital erythrocytosis. (prospective study) Gordeuk et al. [106]	- CP pts and matched controls (*n* = 155). - 40 thrombotic events in CP at enrollment (*n* = 27) with 37 new events (*n* = 33) including 9 fatal events during 11-year observation. Thrombosis in 3 controls at enrollment with 5 new events. - In multivariate analysis in CP pts, age and past thrombosis but not hematocrit were independent predictors of new events. Phlebotomy associated with increased thrombosis (HR 1.9, *p* = 0.028). None were on anticoagulation at second event; aspirin 75 mg/day was not protective. - HIF2A p.M535V variant six-generation pedigree (8 subjects), arterial/venous thrombosis in 5 of 8 vs none in 17 HIF2A wild-type pts (*p* = 0.001). - Thrombotic events despite phlebotomy with HCT < 45%.

On the other hand, phlebotomy might be detrimental to patients with COPD or in adults with cyanotic heart disease and CP, in terms of thrombosis risk [103–106]. Therefore, the institution of phlebotomy in such cases requires careful assessment of risk-benefit balance. The same is true for most cases of secondary erythrocytosis. Moreover, transient symptom relief from phlebotomy is often complicated by a vicious cycle precipitated by depletion of iron stores, resulting in inhibition of PHD2, stabilization of HIF, and increased Epo mediated erythropoiesis. Furthermore, rigid microcytic red cells that are associated with phlebotomy-induced iron deficiency have an inherent tendency to exacerbate hyper-viscosity. Taken together, phlebotomy should be reserved for relief of symptoms with documented response to the particular treatment modality; reported symptoms in patients with non-clonal erythrocytosis include fatigue, generalized weakness, headaches, visual changes, mental fog, tinnitus, chest pain, palpitations, dyspnea, abdominal and bone pain. Similar symptoms can be seen with phlebotomy-induced dehydration and iron deficiency, and therefore should be concomitantly addressed. Aspirin 81 mg daily should be instituted in the presence of cardiovascular risk factors. Arterial and venous thromboses are managed with antiplatelet agents and systemic anticoagulation respectively.

### Management specifics in patients with hypoxic pulmonary disease, including COPD

The incidence of erythrocytosis in COPD ranges from 5.9 to 18.1% and is declining with the implementation of long-term oxygen therapy [107, 108]. Patients are often referred to hematology to address risks for thrombosis and optimal Hct control. Amongst 86 COPD patients with erythrocytosis, retrospectively compared with age- and sex-matched COPD patients without erythrocytosis, no difference in the incidence of venous thromboembolism was recorded: 19.8%vs 14%; *p* = 0.42 [101]. Another study investigated the impact of phlebotomy on prevalence of arterial and venous thrombosis in COPD with erythrocytosis and found similar thrombosis rates in phlebotomized (31%) vs non-phlebotomized (22%) patients (*p* = 0.28) [109]. Moreover, amongst phlebotomized COPD patients, stringent Hct control did not appear to influence the incidence of thrombosis; 25% with Hct <52% vs 37% at higher Hct levels (*p* = 0.45) [109]. These observations are supportive of restricting phlebotomy in COPD to only those patients whose symptoms can clearly be correlated to the increased Hct with documentation of symptom relief from phlebotomy [110]. Furthermore, smoking cessation counseling should be provided when applicable. Management of obstructive sleep apnea (OSA) includes referral to a sleep specialist for institution of overnight continuous positive airway pressure. It is to be noted that in a large study of 1604 patients with suspected OSA, only 1.6% had erythrocytosis defined by Hct ≥51% and 48% in males and females, respectively [111].

### Management specifics in adults with cyanotic congenital heart disease

A retrospective study on 162 adults with erythrocytosis in association with cyanotic heart disease, reported a higher incidence of cerebrovascular events in patients treated with phlebotomy. In that particular study, patients were stratified into two groups based on absence/presence of prior cerebrovascular events; Group 1 (*n* = 140) and Group 2 (*n* = 22), respectively. Amongst 46/162 (28%) of patients that underwent phlebotomy, cerebral events occurred in 25% of Group 1 (*n* = 35) vs 50% of Group 2 (*n* = 11), (*p* = 0.016). Other noteworthy findings were a strong association of iron deficiency and/or microcytosis with cerebrovascular events; reported in 11/41(27%) with iron deficiency; *p* = 0.004. As a result, phlebotomy with volume replacement should be used sparingly for mere symptom relief only when Hct rises above 65%. We also encourage collaborative care with an adult congenital cardiologist [112]. In terms of alternatives to phlebotomy, when 4 patients with symptomatic secondary erythrocytosis and congenital heart disease were treated with hydroxyurea, symptom relief was achieved but without a clear impact on thrombosis risk and at the cost of myelosuppression requiring dose reduction in three patients [113].

### Management specifics in post-renal transplant erythrocytosis

Incidence of erythrocytosis after renal transplant ranges from 8 to 15% and has been associated with increased risk of thromboembolic events [114]. The mechanism remains obscure but excessive Epo production may be driven by angiotensin-II [115]. Accordingly, both ACE inhibitors and angiotensin receptor blockers have been used to control post-renal transplant erythrocytosis, obviating the need for intermittent phlebotomy. The use of enalapril in post-transplant erythrocytosis was studied in a randomized fashion with administration of either 2.5 mg of enalapril daily (*n* = 15) or placebo (*n* = 10) for 4 months. Hct decreased from 52.7 to 47.1 at 1 month and to 46.1 after 4 months of therapy with enalapril; no change with placebo (*p* = 0.004) without any thrombotic events [116]. Similarly, both enalapril and losartan were also evaluated in a prospective randomized study, and both drugs showed a significant decline in Hgb [117]. Theophylline is another agent, which significantly reduced Hct and Epo levels in renal transplant patients (58– 46%; 60–9 units; *p* < 0.05) vs controls (43–39%; 6.9–4.7 units; *p* < 0.05), thereby eliminating requirement of weekly phlebotomy [118].

### Management specifics in patients on androgen therapy

Erythrocytosis may develop in men with androgen deficiency receiving testosterone therapy [119, 120]. Androgen replacement is generally not advised when Hct is >50%. In patients with Hct above 54%, testosterone is withheld until Hct is at an acceptable level below 50%, and therapy resumed at a reduced dose [121]. Amongst 234 transgenders receiving testosterone, with mean Hgb 13.5 g/dL and Hct 40.3% (pre-testosterone), Hgb peaked at 15.7 g/dl, Hct 47.2% at an average of 21 months post therapy [122]. Approximately a quarter of patients developed Hct >50%, one thrombotic event recorded, and dose reduction was implemented in 14.5% of patients with erythrocytosis [122].

### Management specifics in patients with hereditary erythrocytosis

Amongst hereditary conditions associated with erythrocytosis, CP is the only one with a well-defined natural history. It is phenotypically characterized by low systemic blood pressure, body mass index, and white count, with an unusual propensity for vascular events leading to early mortality from both cerebrovascular events and peripheral thrombosis [46]. In a prospective study of 155 CP patients, control-matched by age, sex, and place of residence, the rate of new thrombotic events was considerably higher in CP (37 arterial and venous events of which 9 were fatal) vs controls (5 arterial events) [106]. On multivariate analysis, age and previous thrombotic event, but not Hct, were independent predictors of new events. Moreover, phlebotomy was associated with increased incidence of thrombosis (HR 1.9, *p* = 0.028). In the particular study, none of the patients were on systemic anticoagulation while approximately half were on aspirin therapy at the time of the second event; however, aspirin 75 mg/day was not protective [106].

In addition, in a comparison of CP and PV patients; overall mortality was higher (47%) in CP vs 18.5% with PV, with a higher proportion of deaths due to cerebrovascular events or peripheral thrombosis (46.1% vs 21.9%), regardless of younger age (median age; 16 vs 60 years) [46]. In the particular study, 78% of CP patients received phlebotomy; with no significant benefit in thrombotic risk or mortality reduction, suggesting that thrombotic events are likely driven by factors beyond the high Hct, such as elevated vascular endothelial growth factor, plasminogen activator inhibitor- 1, 2 (PAI-1,2) and homocysteine levels [46]. In regards to drug therapy, in murine models administration of a highly selective JAK2 inhibitor, TG101209 was able to reverse the CP phenotype [48]. To date, three patients have been treated with ruxolitinib with each experiencing hematologic and symptomatic improvement, however, no impact on thrombotic events or mortality has been appreciated [123]. Based on the above, CP patients require close monitoring with stringent cardiovascular risk modification and antiplatelet therapy.

Meanwhile, in a recent prospective observation of eight patients that were part of a six-generation pedigree harboring the *HIF2A* p.M535V variant, arterial and venous thrombosis occurred in 5 of 8 patients vs no events in 17 *HIF2A* wild-type patients (*p* = 0.001). Moreover, thrombotic events occurred despite strict phlebotomy with Hct maintained below 45%, in the absence of cardiovascular risk factors [106].

## Concluding remarks

The discovery of *JAK2* exons 14 and 12 mutations in 2005 and 2007, respectively, and their almost invariable association with PV has greatly simplified our current diagnostic approach to erythrocytosis [73, 124]. This is because the most treatment-relevant step in addressing the differential diagnosis of erythrocytosis is the exclusion of PV, because of its specific association with increased risk of thrombosis and fibrotic/leukemic transformation. The increased utilization of peripheral blood *JAK2* mutation screening has increased general awareness and recognition of non-PV erythrocytosis. In the current document, we have provided practical algorithms for both diagnosing and treating this condition. In this regard, the prompt and accurate distinction between underlying causes is decidedly crucial, as management is directly dictated by etiology, and incorrect diagnosis may lead to *under* or *overtreatment*. Promising discoveries within the last 3 years have included mutations in the *EPO* gene, novel zinc finger domain *PHD2* mutations and splicing mutations in *VHL* [44, 45, 60]. Also, in the foreseeable future, we anticipate improved molecular characterization of the elusive entity, “idiopathic erythrocytosis” [7]. Finally, prospective controlled studies are needed to confirm or further advance current treatment strategies in non-PV erythrocytosis.
